# Genome Wide Analysis of Narcolepsy in China Implicates Novel Immune Loci and Reveals Changes in Association Prior to Versus After the 2009 H1N1 Influenza Pandemic

**DOI:** 10.1371/journal.pgen.1003880

**Published:** 2013-10-31

**Authors:** Fang Han, Juliette Faraco, Xiao Song Dong, Hanna M. Ollila, Ling Lin, Jing Li, Pei An, Shan Wang, Ke Wei Jiang, Zhan Cheng Gao, Long Zhao, Han Yan, Ya Nan Liu, Qing Hua Li, Xiao Zhe Zhang, Yan Hu, Jing Yu Wang, Yun Hui Lu, Chang Jun Lu, Wei Zhou, Joachim Hallmayer, Yu Shu Huang, Kingman P. Strohl, Thomas Pollmächer, Emmanuel Mignot

**Affiliations:** 1 Department of Pulmonary, Critical Care Medicine, Peking University People's Hospital, Beijing, China; 2 Stanford University Center for Sleep Sciences, Palo Alto, California, United States; 3 Department of Surgery, Peking University People's Hospital, Beijing, China; 4 Department of Pulmonary Medicine, Bin Zhou Medical University, Shandong, China; 5 Department of Pulmonary Medicine, Yun Nan Province Hospital, Yun Nan, China; 6 National Taiwan University, Taiwan, China; 7 Division of Pulmonary, Critical Care and Sleep Medicine, Department of Medicine, Case Western Reserve University, and Cleveland Louis Stokes VA Medical Center, Cleveland, Ohio, United States; 8 Center of Mental Health, Ingolstadt, Klinikum Ingolstadt, Krumenauerstrasse, Ingolstadt, Germany; University of Oxford, United Kingdom

## Abstract

Previous studies in narcolepsy, an autoimmune disorder affecting hypocretin (orexin) neurons and recently associated with H1N1 influenza, have demonstrated significant associations with five loci. Using a well-characterized Chinese cohort, we refined known associations in TRA@ and P2RY11-DNMT1 and identified new associations in the TCR beta (TRB@; rs9648789 max P = 3.7×10^−9^ OR 0.77), ZNF365 (rs10995245 max P = 1.2×10^−11^ OR 1.23), and IL10RB-IFNAR1 loci (rs2252931 max P = 2.2×10^−9^ OR 0.75). Variants in the Human Leukocyte Antigen (HLA)- DQ region were associated with age of onset (rs7744020 P = 7.9×10^−9^ beta −1.9 years) and varied significantly among cases with onset after the 2009 H1N1 influenza pandemic compared to previous years (rs9271117 P = 7.8×10^−10^ OR 0.57). These reflected an association of DQB1*03:01 with earlier onset and decreased DQB1*06:02 homozygosity following 2009. Our results illustrate how genetic association can change in the presence of new environmental challenges and suggest that the monitoring of genetic architecture over time may help reveal the appearance of novel triggers for autoimmune diseases.

## Author Summary

Narcolepsy-hypocretin deficiency results from a highly specific autoimmune attack on hypocretin cells. Recent studies have established antigen presentation by specific class II proteins encoded by (HLA DQB1*06:02 and DQA1*01:02) to the cognate T cell receptor as the main disease pathway, with a role for H1N1 influenza in the triggering process. Here, we have used a large and well-characterized cohort of Chinese narcolepsy cases to examine genetic architecture not observed in European samples. We confirmed previously implicated susceptibility genes (T cell receptor alpha, P2RY11), and identify new loci (ZNF365, IL10RB-IFNAR1), most notably, variants at the beta chain of the T cell receptor. We found that one HLA variant, (DQB1*03:01), is associated with dramatically earlier disease onset (nearly 2 years). We also identified differences in HLA haplotype frequencies among cases with onset following the 2009 H1N1 influenza pandemic as compared to before the outbreak, with fewer HLA DQB1*06:02 homozygotes. This may be the first demonstration of such an effect, and suggests that the study of changes in GWAS signals over time could help identify environmental factors in other autoimmune diseases.

## Introduction

A remarkable feature of narcolepsy is its strong HLA association, with similar effects across different ethnicities and countries [1]–[4]. Almost all (98%) cases carry the HLA DQA1*01:02-DQB1*06:02 haplotype, expressing a functional DQα/DQβ heterodimer denoted as DQ0602. Susceptibility is further increased in DQB1*06:02 homozygotes [5], and DQB1*06:02/DQB1*03:01 heterozygotes [1]–[3]. It is also lower in subjects with HLA DQA1*01:02-DQB1*06:02 and other, non-DQA1*01:02 and DQB1*06:02 DQ1 alleles [1]–[4], an effect likely due to trans-dimerization and reduction of DQ0602 availability [3]. Genome wide association studies (GWAs) of individuals of European ancestry have identified TRA@, P2RY11-DNMT1, CTSH and TNFSF4 loci as additional susceptibility genes [6]–[8].

Recently, a strong link between upper airway winter infections and narcolepsy has emerged. Yearly patterns of narcolepsy onset in China revealed a ∼6 fold increase in spring and summer versus winter [9]. Associations between group A Streptococcus Pyogenes and narcolepsy have been found in several studies [10]–[12]. Following a 2009 pandemic H1N1 (pH1N1) vaccination campaign in Europe, increased risk linked to Pandemrix exposure, an ASO3 adjuvanted vaccine formulation, was reported in multiple countries [13]–[17], raising alarm. Incidence in China sharply increased 4 months after the 2009 H1N1 influenza pandemic peak, returning to previous rates following the pandemic [9], [18]. All these cases are HLA DQB1*06:02 positive, and have hypocretin deficiency when documented [12], [19]. The fact pH1N1 was practically unknown to humans prior to late 2009 [20] offers a unique opportunity to understand how pathogens are involved in triggering autoimmune diseases.

To identify novel narcolepsy susceptibility loci potentially missed in previous studies focused on European ancestry, we studied 1,189 Chinese narcolepsy cases primarily characterized at a single clinical center (Beijing University) [9], [18], [21] and 1,997 Chinese controls genotyped on the Affymetrix Axiom CHB array. All cases had documented hypocretin deficiency or had clear-cut cataplexy and HLA DQB1*06:02, ensuring etiological homogeneity and meeting ICSD3 criteria for type 1 narcolepsy. We tested allelic association at 603,382 non-HLA, autosomal SNPs, correcting for stratification using a mixed model method (inflation statistic, lambda = 1.001).

## Results and Discussion

Genome wide significant association signal (GWAS, p≤5×10^−8^) was seen for 9 SNPs in the TRA@ locus (Figure S1). We selected the top 80 nominally significant SNP loci for replication testing or combined analysis (see methods) in narcoleptics from two European cohorts typed on the Illumina ImmunoChip (1886 cases, 10,421 controls) and Affymetrix 6.0 arrays (807 cases, 1074 controls) [6], [8], (Table S1). These cohorts had partially overlapping cases, thus we first attempted replication in the ImmunoChip dataset, which has a larger sample size but covers only selected immune-related loci; when this was not possible, we used the smaller Affymetrix 6.0 dataset. Two of the 5 SNPs that were testable in the ImmunoChip dataset were significant after Bonferroni correction: rs1154155 at TRA@ (p_Chinese_ = 6.32×10^−20^,p_Eur_ = 8.87×10^−30^), and rs10995245 at ZNF365 (p_Chinese_ = 2.34×10^−4^,p_Eur_ = 3.24×10^−7^). Thirty-eight additional variants were testable using the Affymetrix 6.0 data set, with confirmation of the PPAN-P2RY11-DNMT1 region (rs1551570, p_Chinese_ = 1.88×10^−6^,p_Eur_ = 5.10×10^−8^), and new associations identified at rs2834188 on Chromosome 21 at the IL10RB-IFNAR1 locus (p_Chinese_ = 1.78×10^−4^,p_Eur_ = 5.92×10^−5^, and rs2853536 on Chromosome 7 at the T cell receptor beta locus (TRB@, p_Chinese_ = 7.20×10^−5^,p_Eur_ = 9.44×10^−4^), both strong biological candidates (Table 1). To further characterize these associations, we imputed genotypes surrounding these five loci in Chinese and Europeans, and performed combined regional associations. The combined association studies yielded genome-wide significant values at each of the 5 loci (Table 1).

**Table 1:** Replicated association and combined analysis at five SNP loci.

				Chinese discovery cohort				European data sets						Combined analysis	
				Affymetrix CHB						ImmunoChip		Affymetrix 6.0		Mantel Haenszel test	
Chr	SNP	Locus	Effect allele	Freq Chinese controls	Freq Chinese cases	P Value	OR	Freq European controls	Freq European cases	P Value	OR	P Value	OR	P Value	OR
14	rs1154155	TRA@	G	0.510	0.628	6.32×10^−20^	1.61 (1.47–1.79)	0.148	0.229	8.87×10^−30^	1.72 (1.54–1.90)	NA***		5.02×10^−49^	1.64 (1.53–1.75)
19	rs1551570	P2RY11	C	0.305	0.248	1.88×10^−6^	0.75 (0.67–0.84)	0.542*	0.608*	NA		5.19×10^−8^ *	0.75 (0.68–0.83)*	3.77×10^−10^	0.76 (0.70–0.83)
7	rs2854536	TRB@	G	0.256	0.212	7.20×10^−5^	0.79 (0.70–0.89)	0.708	0.652	NA		9.44×10^−4^	0.77 (0.66–0.90)	3.87×10^−8^	0.78 (0.71–0.85)
21	rs2834188	IL10RB-INFAR1	A	0.296	0.2525	1.78×10^−4^	0.80 (0.72–0.90)	0.295**	0.236	NA		5.92×10^−5^ **	0.74 (0.64–0.86)**	1.95×10^−8^	0.77 (0.71–0.85)
10	rs10995245	ZNF365	A	0.287	0.3346	2.34×10^−4^	1.25 (1.12–1.40)	0.348	0.390	3.24×10^−7^	1.22 (1.09–1.31)	NA	NA	1.24×10^−11^	1.23 (1.16–1.31)

Chr: Chromosome; OR:Odds Ratio; NA:not applicable or not available; European Data sets: previously published ImmunoChip and Affymetrix 6.0 cohorts.

P values for Affymetrix CHB and ImmunoChip study were calculated with EMMAX, P value for Affymetrix 6.0 and Combined analyses of all cohorts were calculated with Mantel Haenszel test.

* p value, allele frequency, and OR displayed for proxy SNP rs2305795 (minor allele = A, r2 = 0.9) from Kornum et al.

** p value, allele frequency, and OR displayed for proxy SNP rs2409488 (minor allele = A, r2 = 1);

*** See reference 6 Hallmayer et. al.

**Figure 1:**
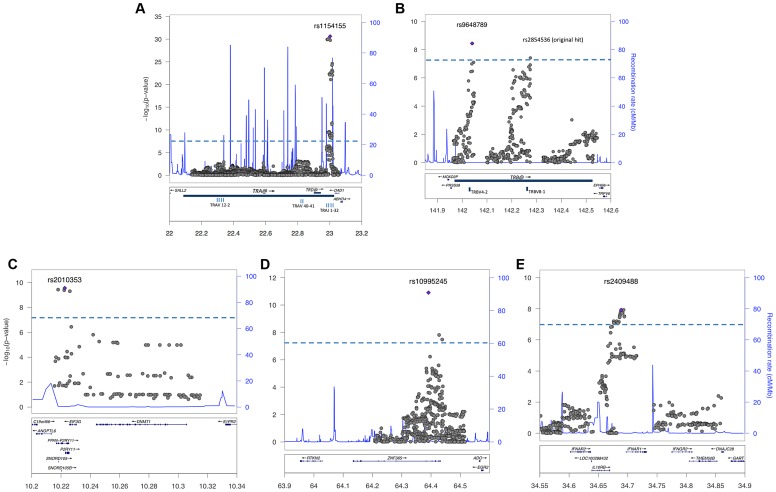
Regional analysis of genome-wide significant loci. Combined association scores were computed using Mantel Haenszel X^2^ test, following imputation of genotypes surrounding replicated SNPs in Chinese and European samples. The top scoring SNP for each region is marked with a purple diamond. Strength of LD is not indicated, as results represent data from two ethnic groups. The X-axis scale shows chromosomal position (Mb) from human genome reference sequence (hg19). The left Y-axis shows the negative base ten logarithm of the p-value, with genome-wide significance threshold (P<5×10^−8^) marked by dashed blue line. The right Y-axis shows recombination rate (cM/Mb) as a blue line. Genes in the regions are annotated at the bottom as blue bars. A: T cell receptor alpha on chromosome 14; B. T cell receptor beta on chromosome 7; C. P2RY11-DNMT1 on chromosome 19; D. ZNF365 on chromosome 10; E. IL10RB-INFAR1 region on chromosome 21.

Consistent with previous reports [6], [8], [22], the large TRA@ locus encoding the α-chain of the T-cell receptor, showed the strongest association with narcolepsy. Combining the present sample with the large sample typed on the ImmunoChip yielded a p value of 5.0×10^−49^ at rs1154155G (OR 1.6), but low coverage on that array prevented fine mapping. This variant is also the most significant (P = 2.3×10^−31^, OR 1.7) among 35 SNPs when the Chinese and European Affymetrix 6.0 data sets are combined. The most highly significant variants cluster within a 22 kb region encompassing the TRAJ segments J8 through J28. (Figure 1A). Of special interest as a candidate causal SNP is rs1483979, located within the J24 segment and projected to change an aminoacid (F8L) within the CDR3 peptide-binding site of any TCR carrying J24. Linkage Disequilibrium (LD) between rs1483979C and rs1154155G is high in both populations (r2≥0.8).

Complementing the TRA@ association, we found associations at three variants within the T cell receptor beta locus (TRB@), which encodes the β-chain of the T-cell receptor. A first association at rs2854536 (3.87×10^−8^, OR 0.78), near the pseudogene TRBV8-1, reached genome wide significance, extending on a tentative finding in a prior study [6]. In addition, we found an even higher independent association at 2 tightly linked SNPs, rs9648789T and rs3020837A (both 3.7×10^−9^, OR 0.77 pairwise r^2^ = 1) that are unlinked with rs2854536 (r^2^ = 0.21) and encompass an area of approximately 14 kb containing the TRBV7-1 and TRBV4-2 segments (Figure 1B). Inspection of 1000genomes did not reveal candidate coding SNPs for these segments, suggesting more complex effects, for example on recombination and Vβ usage. Like antibody genes, T cell receptor loci undergo somatic recombination, thus most of the diversity is not encoded at the genomic level. While the TRA@ locus contains only V, J and C segments, the TRB@ locus also includes two D segments, dramatically increasing repertoire diversity, and likely obscuring genomic associations in smaller, less powerful samples. The fact that associations with these loci are found in narcolepsy but not other HLA-associated diseases may reflect a relative oligoclonality of the TCR subtypes involved in the autoimmune process, itself tightly associated with a single DQα/β heterodimer.

Our study also extends on a previously reported association within the purinergic receptor P2RY11 gene [7], [22] at rs2010353G (P = 2.8×10^−10^, OR 0.76) in intron 1 (Figure 1C). Four SNPs with tight LD in both Chinese and European populations are representative of this association, which does not extend to the DNMT1 locus, where the association signal is low. This supports our previous results implicating this gene, which mediates survival of T lymphocytes and NK cells in response to ATP-induced cell death, in the pathogenesis of narcolepsy ([7]). Of interest, rare mutations in the neighboring DNMT1 locus cause narcolepsy together with ataxia, deafness and dementia [23]. The present results do not support a hypothesis that the P2RY11 association reflects the action of a causative variant within the DNMT1 locus, although this conclusion is limited by available SNPs present on the two chip arrays and their ability to tag any such putative variants. Further, elements located within the P2RY11 may regulate DNMT1 expression. The P2RY11-DNMT1 gene segment is conserved in zebrafish, suggesting co-evolutionary pressures.

ZNF365, a gene associated with multiple phenotypes including breast cancer, sudden cardiac death from coronary disease, Crohn's disease, and atopic dermatitis [24]–[27] already had suggestive association in the ImmunoChip study of narcolepsy in European ancestry [8] and was strongly replicated in the Chinese cohort, reaching GWA-significance at rs10995245A in the combined sample (P = 1.2×10^−11^, OR 1.23) (Figure 1D). Two additional variants in moderate LD in both populations also surpassed this threshold. The gene has high levels of expression in the brain and spans nearly 300 kb, encoding 10 transcripts and several isoforms encoding distinct proteins with different expression patterns and functions. The narcolepsy variant is not linked to coding SNP rs7076156 (Ala62Thr) implicated in Crohn's disease [28] (r2 = 0.166 in Europeans).

A last locus, encompassing the IL10RB-IFNAR1 genes, also replicated strongly and reached GWAS significance in the combined sample. SNPs rs2409488A and rs2834190T, located between IL10RB and IFNAR1, were most significant (both P = 1.2×10^−8^ OR 0.76) although a total of 13 variants reached GWA significance (Figure 1E). The two loci are located within a cluster of class II cytokine receptor genes on chr21q22 and are separated by 27 kb. The segment is within a region of high LD covering most of IL10RB gene through the 3′ end of the IFNAR1 gene. Both genes are strong candidates for narcolepsy. The IL10RB gene encodes a chain shared by several receptors of the IL10 cytokine family (an anti-inflammatory cytokine), and is associated with Crohn's disease both in GWA studies and in rare multiplex families with early onset Inflammatory Bowel Disease [29], [30]. IFNAR1 encodes the α-chain of the IFN-α/β receptor, and is another appealing functional candidate, as IFN-α/β signaling is not only an early response to viral infection, but IFNAR1 null mice demonstrated more severe autoimmune disease of the central nervous system in a model of experimental autoimmune encephalomyelitis [31].

We also examined variants previously reported to be associated with narcolepsy. It was not possible to replicate associations at the TNFSF4 and CTSH loci [8] in the Chinese cohort due to a lack of markers in high LD with previously associated variants (rs7553711 at TNFSF4, or rs3843303 and rs34593439 at CTSH) on the array. There was no association around CPT1B-CHKB (rs5770911 proxy r2 = 1 with rs5770917, P = 0.66, OR 1.02), consistent with previous findings [22]. Overall, this study brings the number of GWA significant loci for narcolepsy to 8, further implicating the immune system notably antigen presentation by HLA-DQ to the TCR as the primary cause for the disorder. None of the explored loci showed significant pairwise interactions, including those involving HLA and the T-Cell receptor (data not shown).

We next examined genetic associations with specific clinical characteristics within the cohort, starting with previously associated loci and extending to genome-wide analyses. One of the unique features of the Chinese cohort is the large sample size with phenotype data consistently collected using the same procedure for over 10 years. The majority of these cases are children and diagnostic delay was short (5 years) compared to Europe (15 years) [32], likely improving recollection (see methods). Clinical characteristics that were available on ≥1000 cases were examined, including age of onset (cataplexy, sleepiness or the earliest of either) and sleep test results. Because narcolepsy incidence increased 6 fold following the 2009 influenza pandemic, we also compared cases with onset before versus after September 2009.

**Figure 2:**
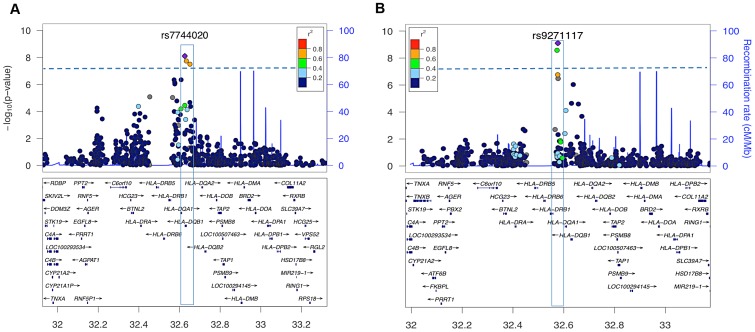
HLA-region SNP markers associated with clinical presentation at genome-wide significant levels. Plots depict genotyped SNPs in the extended HLA region of chromosome 6 with top ranking SNPs marked as purple diamonds. The X-axis shows position on chromosome 6 from human genome reference sequence (hg19), the Y-axis (left) negative base ten logarithm of p-value, the Y-axis (right) recombination rate (cM/Mb) as a blue line. Pairwise LD (r2) is color coded according to strength in 1000 genomes Asian populations. The genome-wide significance threshold (P = 5×10^−8^) is given by the dashed blue line. A: Association signal of genotyped SNPs in a quantitative trait association of age of onset among cases with onset between 2–33 years of age (41 cases excluded as outliers). Three variants near DQB1 reached significance, the highest value being at rs7744020 (Table 2). This variant was subsequently shown to be most tightly associated with DQB1*03:01 (see Table S2). B. Association statistics comparing cases with onset before (N = 726) vs after (N = 251) September 2009. Two HLA SNP markers were genome wide significant, rs9271117 and rs9270965. These variants were subsequently shown to be most tightly, but not exclusively, associated with DQA1*01:02 (see Table S2).

Genome-wide significant effects were observed for multiple SNPs in the HLA-DQ region for both age of onset (rs7744020 P = 9.0×10^−9^ Beta −1.9 years, SE 0.33), and among cases with onset after the 2009 H1N1 pandemic versus prior years (rs9271117 P = 6.7×10^−14^ OR 0.57) (Figure 2, Table 2). The association was not due to population stratification as cases pre and post 2009 did not differ in their geographic distribution or principal components (Figure S2 and S3). Among 685 cases with onset ≤10 years, rs7744020A had a frequency of 0.24, compared to 0.14 in 155 cases with onset ≥15 (P = 0.0003, OR 1.88). No other significant associations were observed with other characteristics, including for rs12322530 and cataplexy onset (1069 individuals cataplexy onset age 2–55; P = 0.99, beta 0.01, SE 0.69), a proxy for rs12425451 (r2 = 0.94) that was nominally associated with cataplexy onset in a European cohort [32]. None of the identified GWA significant loci showed significant interactions with clinical variables.

**Table 2:** SNPs surpassing GWA significance in disease presentation studies, and their associated HLA alleles.

Genome-wide analysis in cases						Association of SNPs with HLA subtypes in cases			
SNP	Trait	P value (EMMAX)	P value (Plink)	Allele frequency	Effect size (beta, years)	HLA allele	Frequency in haplotypes with DQB1*03:01 (N)	Frequency in haplotypes without DQB1*03:01 (N)	P Value
rs9274477G	Age of disease onset	2.09×10^−8^	1.83×10^−8^	0.292	−1.82	DQB1*03:01	0.69 (512)	0.035(1861)	5.61×10^−264^
rs17212223T	Age of disease onset	7.73×10^−8^	3.17×10^−8^	0.191	−1.95	DQB1*03:01	0.99 (360)	0.033 (2013)	0.00
rs7744020A	Age of disease onset	9.00×10^−9^	7.89×10^−9^	0.272	−1.9	DQB1*03:01	0.73 (487)	0.035(1886)	7.46×10^−281^

Genome-wide analysis in cases						Association of SNPs with HLA subtypes in cases			
SNP	Trait	P value (EMMAX)	P value (Plink)	Allele frequency (N)	Effect size (OR)	HLA allele	Frequency of SNP in haplotypes with HLA allele (N)	Frequency of SNP in haplotypes without HLA allele (N)	P Value
rs9270965A	Narcolepsy after vs. before 2009	4.96×10^−12^	2.67×10^−9^	Before: 0.630 (893) After: 0.497 (373)	0.58	DQA1*01:01	0.31 (48)	0.58 (2324)	2.66×10^−04^
						DQA1*01:02	0.88 (1401)	0.12 (971)	1.04×10^−302^
						DQA1*01:03	0.56 (39)	0.57 (2333)	0.94
						DQB1*06:4 or 06:09	0 (34)	0.58 (2338)	1.31×10^−11^
						DQA1*05:08	0.30 (23)	0.57 (2349)	9.59×10^−03^
						DQB1*05:01	0.58 (24)	0.57 (2348)	0.9
						DQB1*05:02	0.75 (83)	0.56 (2289)	9.39×10^−04^
						DQB1*06:01	0.79 (82)	0.56 (2293)	3.31×10^−05^
						DQB1*06:02	0.90 (1256)	0.19 (1116)	2.35×10^−266^
rs9271117C	Narcolepsy after vs. before 2009	6.38×10^−14^	7.89×10^−10^	Before: 0.605 (874) After: 0.468 (355)	0.57	DQA1*01:01	0.31 (48)	0.55 (2324)	1.08×10^−3^
						DQA1*01:02	0.89 (1401)	0.05 (971)	0.00
						DQA1*01:03	0.59 (39)	0.54 (2333)	0.57
						DQB1*06:4 or 06:09	0 (34)	0.55 (2338)	1.28×10^−10^
						DQA1*05:08	0.30 (23)	0.55 (2349)	0.02
						DQB1*05:01	0.58 (24)	0.54 (2289)	0.71
						DQB1*05:02	0.77 (83)	0.54 (2289)	1.70×10^−4^
						DQB1*06:01	0.80 (82)	0.54 (2290)	1.53×10^−6^
						DQB1*06:02	0.91 (1256)	0.14 (1116)	0.00

N = number of subjects or haplotypes, whichever is applicable.

SNP associations within the HLA are difficult to interpret, as specific SNPs may be associated with multiple haplotypes, creating inflated or deflated p values that may not have a simple interpretation. We therefore examined the effects of specific HLA alleles carried by our cases on the clinical presentation. In narcolepsy, HLA-DR and DQ associations are established, and the fact nearly all patients carry DQA1*01:02-DQB1*06:02 on at least one chromosome facilitates interpretation and imputation. To study HLA allele effects, we imputed HLA-DQ in our sample using a reference set of 239 cases and 14 controls (partially described in [3]). All reference individuals carried DQB1*06:02 and were fully HLA-DQA1 and DQB1 typed. Further analyses on the effects of specific HLA alleles in our sample were then performed only on individuals carrying least one copy of DQB1*06:02 (1183 cases and 438 controls) to avoid issues pertaining to differential imputation quality in cases versus controls (who carry a more diverse array of HLA alleles). Using this method, DR and DQ allele frequencies in cases versus controls were compiled, and showed expected effects (Table 3, Table S3). We next categorized the sample into 5 subgroups based on established relative risk categories. Consistent with prior reports, DQB1*06:02 homozygotes carried the highest risk, followed by DQB1*06:02/DQB1*03:01 heterozygotes (Table 3). In contrast, individuals carrying non-DQA1*01:02, non-DQB1*06:02-DQ1 alleles were rather protected as predicted by the allele competition model [3]. These results establish validity of our imputed HLA data.

**Table 3:** Analysis of predisposing and protective HLA allelic combinations in Chinese individuals that carry DQB1*06:02.

HLA allele risk category	Narcolepsy				Age of onset (2–33) in years	
	N Case (freq)	N Control (freq)	P	OR (CI)	P	Beta (SE)
DQB1*06:02 homozygous	0.13 (151)	0.06 (26)	3.72×10^−4^	2.24 (1.46–3.45)	0.006	1.31 (0.47)
DQB1*03:01 carriers	0.30 (359)	0.27 (116)	0.018	1.35 (1.11–1.74)	8.62×10^−8^	−1.87 (0.35)
DQA1*01:02 with DQB1*05 or DQB1*06, non-06:02	0.13 (150)	0.11 (48)	0.034	1.47 (1.03–2.10)	0.98	0.01 (0.53)
DQA1*01-non*01:02	0.08 (97)	0.19 (82)	6.28×10^−6^	0.46 (0.32–0.63)	0.088	1.16 (0.68)
other	0.36 (430)	0.38 (166)	REF	REF	REF	REF

HLA allele risk category	After/Before 2009					
	N Before (freq)	N After (freq)	P	OR (CI)		
DQB1*06:02 homozygous	0.15 (108)	0.09 (34)	0.003	0.52 (0.35–0.79)		
DQB1*03:01 carriers	0.29 (208)	0.34 (127)	0.333	1.15 (0.87–1.51)		
DQA1*01:02 with DQB1*05 or DQB1*06, non-06:02	0.13 (95)	0.11 (41)	0.201	0.77 (0.51–1.15)		
DQA1*01-non*01:02	0.09 (63)	0.07 (26)	0.138	0.69 (0.42–1.13)		
other	0.35 (251)	0.40 (151)	REF	REF		

The comparison used only DQA1*01:02-DQB1*06:02 positive individuals (1183 cases, 438 controls) thus p values are well-below those reported in Table 2 (see text).

The analysis was done sequentially first analyzing DQB1*06:02 homozygotes, then DQB1*03:01 carriers, then individuals with DQA1*01:02 with non-DQB1*06:02 at the other allele, and finally DQA1*01-nonDQA1*01:02 carriers.

As suggested by the rs7744020 association with this allele (Table 2, Table S2), DQB1*03:01 had a strong effect on earlier age of onset (P = 8.62×10^−8^ Beta −1.87 years; Table 3). This finding is of particular interest, as the predisposing mechanism of DQB1*03:01 is not explained by the allelic competition model, and is independent of DQA1 [1], [2]. The divergent effects of DQB1*03:01 and DQB1*06:02 on age of onset further support a different mechanism of action for this allele, perhaps an effect of T-Cell receptor repertoire. A prior study also found no effect of DQB1*06:02 homozygosity on disease onset in Caucasians [33]. Interestingly, DQB1*03:01 frequency is high in China, and variable across Europe, possibly explaining why an unusually large number of cases with childhood onset are reported in China versus US and Europe.

Table 3 also describes overall HLA association across HLA genotype risk categories for cases with onset after versus prior to the 2009 H1N1 pandemic. Consistent with the genome wide significant effect of rs9271117, a marker located between DQA1 and DRB1 and mostly linked with DQA1*01:02 (Table 2, Table S2), fewer DQB1*06:02 homozygotes were found in subjects with disease onset following the influenza H1N1 pandemic in China (P = 0.003 OR 0.52; Table 3, Table S3). In contrast, DQB1*03:01 had no effect (Table 3). Although these results suggest HLA differences in subjects with onset prior versus after 2009, the effect of rs9271117 (Table 2) was stronger than the effects of DQB1*06:02 homozygocity (Table 3) and of all other individual HLA effects we could impute (Table S3). It was also independent of DQB1*03:01 and of age of onset differences, remaining genome wide significant after controlling for these factors (data not shown). This finding is unique as, to our knowledge it is the first time a GWA significant signal has been shown to vary with calendar time, a variable rarely considered in existing GWAS of autoimmune diseases. The 2009 H1N1 pandemic was a remarkable event, as although sporadic cases of swine to human infections were reported with similar swine flu strains as early as 1998, only in 2009 did this new variant cross the species barrier [20], transmitting rapidly first in children and young adults in the winter of 2009–2010 [34]. Perhaps post H1N1 cases involve presentation of new epitopes to HLA alleles not identified in prior studies, explaining the differential HLA region association, an hypothesis that will only be answered through full HLA typing, notably of additional DRB genes. Alternatively, a linked variant could have regulatory effects. We hypothesize that GWA analysis of other diseases across time, notably those with an autoimmune component, may help decipher the timing and nature of environmental factors linked to specific disease pathophysiology. This may prove powerful as sample size for these diseases increases, as identification of environmental factors for most diseases has been more resistant to investigation than genetic analysis.

## Materials and Methods

### Subjects

Our sample included 1189 narcolepsy cases, 1136 of whom were seen at the sleep laboratory of People's Hospital, Peking University, Beijing, a unit in the pulmonary medicine department evaluating patients with sleep disorders and receiving referrals from all over China. In addition, 51 samples came from Taiwan (Dr. Huang, National Taiwan University) and two from Stanford. All patients had either documented hypocretin deficiency (CSF hypocretin-1≤110 pg/ml, n = 119), or clear-cut cataplexy and HLA-DQB1*06:02 [6]. Cases were mostly Han descent (87%) and from North China (85%). The majority of our cases were male (67%). A majority of cases are children (70%) [21]; mean age was 11.2±0.2 years (11.6±0.3 in males versus 10.55±0.39 for females). Clinical data included age of disease onset (earliest onset of cataplexy or sleepiness), presence or absence of cataplexy, sleepiness, sleep paralysis, hypnogogic hallucinations, and disturbed nocturnal sleep, and Multiple Sleep Latency Testing data (mean sleep latency and number of sleep onset REM sleep periods). Delay between disease onset and diagnosis was also noted [9] [18]. Mean ± SEM are reported for age of onset, and diagnostic delay. Control genotypes from China came from university employees and students (41% male), and shared controls from GWAS studies underway for colon cancer and Sjogren's syndrome.

### Ethics statement

Informed consent in accordance with governing institutions was obtained from all subjects. The research protocols were approved by IRB Panels on Medical Human Subjects at both Stanford University and the Beijing University People's Hospital.

### Genotyping, quality control and SNP selection

DNA samples were genotyped on the Affymetrix Axiom CHB array. Genotypes were called using Affymetrix Genotyping Console. Individuals with call rate <95%, or that were outliers following principal components analysis (n = 47), or related (n = 53), were removed, leaving 1189 cases and 1997 controls. For the main association study, we selected SNP variants with MAF ≥1%, call rate ≥90%, and HWE p value ≥0.001 in controls. Because of the near requirement for DQB1*06:02 in narcolepsy, an extended HLA region from rs1419229 to rs9368865 (Chr 6:24112537-35363736) was excluded from the primary association, leaving 603,382 autosomal, non-HLA SNP variants. The extended HLA region contained 3,000 SNPs and was analyzed separately for imputation of HLA haplotypes and effects on clinical presentation.

### Association statistics

The majority of the analysis (including quality control, LD calculations, quantitative trait association, and interactions) was performed using the Plink suite of software (v 1.07) [35]. To control for population stratification, the association was performed using a variance component model implemented in EMMAX [36]. Cluster quality of top ranking SNP markers was verified by visual examination of clusters. As EMMAX does not return OR or MAF data, these data were gathered and reported from corresponding analyses in Plink. Inflation statistics were estimated, and QQ plots were generated using estlambda (GenABEL package (v1.7-4) in R (2.15.3) [37] [38]. In addition, Principal Components was performed, and Manhattan plot generated using SVS v7 (GoldenHelix).

### Replication

The top ranked 150 SNPs from associations performed in EMMAX and Plink (a total of 188 SNPs, p≤0.001 for EMMAX, ≤0.003 for Plink) were selected for follow-up. After visual check and exclusion of 16 poorly clustered SNPs, we selected the top-ranked SNP from each locus (defined as 2 SNPs within 100 kb) resulting in 80 variants for replication in our previously published European cohorts (Affymetrix 6.0 array study: 807 narcolepsy, 1071 controls; Illumina Immunochip array study 1886 cases, 10,421 controls). The Affymetrix and Illumina Immunochip samples were partially overlapping and thus the replication was first done in the larger Illumina immunochip sample. If a variant was not present or tagged by variants on that array, then replication was next attempted in the smaller Affymetrix 6.0 data set. When a selected CHB variant was not on the corresponding array, an analysis of LD in a 10–20 kb window was performed in Chinese and Europeans (Ensembl 1000genomes browser LD data) to identify potential proxies with an r2 ≥0.8 available on the ImmunoChip or Affymetrix 6.0 array. In the case of rs1551570, the variant was not tagged on either array, but shows strong LD with rs2305795, a SNP found to be associated with narcolepsy following fine mapping [7] where corresponding p values were extracted from the previous analysis. A Bonferroni correction was applied to determine significance.

### Clinical associations

Multiple Sleep Latency Testing data (mean sleep latency and number of sleep onset REM sleep periods) and age of onset were studied using genome wide linear regressions as quantitative traits (Plink). Genome-wide significant values obtained by Plink were then re-tested using EMMAX, with EMMAX P values reported in Table 2. Presence or absence of cataplexy, sleep paralysis, hypnogogic hallucinations, as well as whether or not onset was prior to September 2009, were studied as binary phenotypes (Plink).

### Imputation and combined association analysis of Chinese and Caucasian data

We imputed genotypes in windows that fully surrounded each of the 5 replicated loci. Imputation was performed separately in the Chinese Axiom CHB, and European ImmunoChip, Affymetrix 6.0 using Beagle v3.3 [39] against the CHB reference Chinese population, or 4 European populations (286 individuals from CEU, TSI, GBR, IBS) in the 1000 genomes integrated data set (phase 1 release v3). Imputation was performed in the Chinese cohort for all five replicated loci (TRA@, TRB@, ZNF365, PPAN-DNMT-1, IL10RB-IFNAR1 region). These loci were also imputed in European ancestry narcolepsy samples depending on regional SNP coverage on the corresponding genotype array (TRA@ in Affymetrix 6.0, with rs1154155 also in Immunochip; TRB@ in Affymetrix 6.0, not covered in ImmunoChip; ZNF365 in ImmunoChip, not covered on Affymetrix 6.0; PPAN-DNMT1 in Affymetrix 6.0, not covered in ImmunoChip, IL10RB-IFNAR1 Affymetrix 6.0- no SNPs in LD with rs2834188 on ImmunoChip). Imputed genotypes were combined (Chinese+ ImmunoChip, or Chinese+Affymetrix 6.0) and associations were performed using a Mantel Haenszel test (Plink). SNP markers with poor imputation quality scores in either Chinese or Europeans (r2<0.8) were excluded from further analysis. Plots of association statistics were made using LocusZoom [40].

### Imputation of HLA haplotypes and alleles in DQB1*06:02 positive subjects

High resolution HLA typing had been performed on a subset of 239 narcolepsy cases and 14 controls for HLA DRB1, DQA1 and DQB1 genes [3], all positive for DQB1*06:02. These individuals were also genotyped on the Axiom CHB array (see above). In order to impute the HLA genotypes for the rest of the data set, the HLA types, together with array-genotyped SNPs in a 500 kb window surrounding HLA DRB1, DQA1 and DQB1 were submitted as a training set to HIBAG package in R. HIBAG is an HLA imputation tool that uses attribute bootstrap aggregation of several classifiers (SNPs) to select groups of SNPS that predict HLA type [41]. The resulting sets of haplotype predictive SNPs were then used to impute HLA type in the remaining samples (cases and controls). Allele frequencies of DRB1, DQA1 and DQB1 alleles obtained after imputation was consistent with population data (r2 = 0.96) [42]. Imputation was acceptable in both DQB1*06:02 positive and negative controls, but in consideration of our training set, we only used data from DQB1*06:02 positive individuals, which was imputed with better quality (85.0% in DQB1*06:02 positive individuals and 77.5% in DQB1*06:02 negative individuals). Imputation of DQB1*03:01, DQB1*06:02 and DQB1*01:02, the alleles of principal interest in this study was highly accurate, (DQB1*03:01: 92.8% in controls 97.6% in cases, DQB1*06:02: 90.5% in controls, 97.4% in cases, DQA1*01:02: 92.2% in controls, 97.5% in cases) in the data set. The imputation quality may be overestimated in cases since the narcolepsy population is more homogenous for their HLA haplotype.

### Sub analyses of DQB1*06:02 positive individuals

The imputed HLA haplotype data was first used to study association between rs7744020, rs9274477, rs17212223 (age of onset associated), rs9271117, rs9270965 (associated with onset prior versus after September 2009) and various HLA genotypes (Table 2). To verify whether the HLA association in narcolepsy versus controls was consistent with previous reports in other ethnic groups [1], including Chinese subjects [3] (N = 1183 DQB1*06:02 positive cases and N = 438 DQB1*06:02 positive controls), we next categorized subjects into five risk groups based on our prior model of allelic competition [3]: 1) DQB1*06:02 homozygous (highest risk); 2) DQB1*03:01 carriers (second highest risk); 3) DQA1*01:02-DQB1*05 or DQB1*06 non-06:02 (intermediate) 4) DQA1*01 non-DQA1*01:02 (protective) 5) alleles with no predisposition or protection effects for narcolepsy. These categories were then compared using χ^2^ square tests between cases versus controls and cases after versus before September 2009. χ^2^ analyses were performed sequentially, starting with DQB1*06:02 homozygotes, then moving on to DQB1*03:01 carriers, then to DQA1*01:02-DQB1*05 or DQB1*06 non-06:02 carriers, and finally to DQA1*01 non-DQA1*01:02 carriers, as previously reported [3], a technique similar to relative predisposition effect statistics [43].

### Interaction analysis

Potential interactions between narcolepsy risk SNPs and HLA genotypes were analyzed with R version 2.15.3, and with an epistasis model Y ∼b0 + b1.A + b2.B + b3.AB +e implemented in Plink. The interaction was performed with narcolepsy, onset after/before 2009 and with age of onset.

### URLs

http://faculty.washington.edu/browning/beagle/beagle.html

http://bochet.gcc.biostat.washington.edu/beagle/1000_Genomes.phase1_release_v3/

http://csg.sph.umich.edu/locuszoom/

http://www.R-project.org/

## Supporting Information

Figure S1 A: Plot of association statistics for 603,382 autosomal, non-HLA variants calculated with EMMAX. The significance threshold used (blue line) was P = 5×10^−8^. The inset depicts a quantile quantile plot of results observed (black circles), and slopes of estimated (red) versus expected chi square values (black line, invisible). The inflation statistic for tested markers is 1.001. B: List of risk variants to significance of P = 5×10^−8^.
(TIF)

Figure S2 Multidimensional scaling plot of first three components in Chinese. The Chinese controls are shown in black, narcoleptics before 2009 shown in green, narcoleptics after 2009 in red and controls from Hapmap 3 in blue.
(TIF)

Figure S3 QQ-plot and Manhattan plot before and after 2009 and North vs. South China and comparison of individuals from the difference provinces in China for onset after vs. before 2009.
(TIF)

Table S1 80 SNP hits selected for replication in European data sets.
(XLSX)

Table S2 Genome-wide significant SNPs in the HLA region tagging HLA haplotypes.
(XLSX)

Table S3 HLA allele and haplotype frequencies.
(XLSX)
