# Supplementary figures and images for: Genome Wide Analysis of Narcolepsy in China Implicates Novel Immune Loci and Reveals Changes in Association Prior to Versus After the 2009 H1N1 Influenza Pandemic
Source: PLoS Genet. 2013 Oct 31;9(10):e1003880. doi: 10.1371/journal.pgen.1003880 (PMC3814311; doi:10.1371/journal.pgen.1003880)

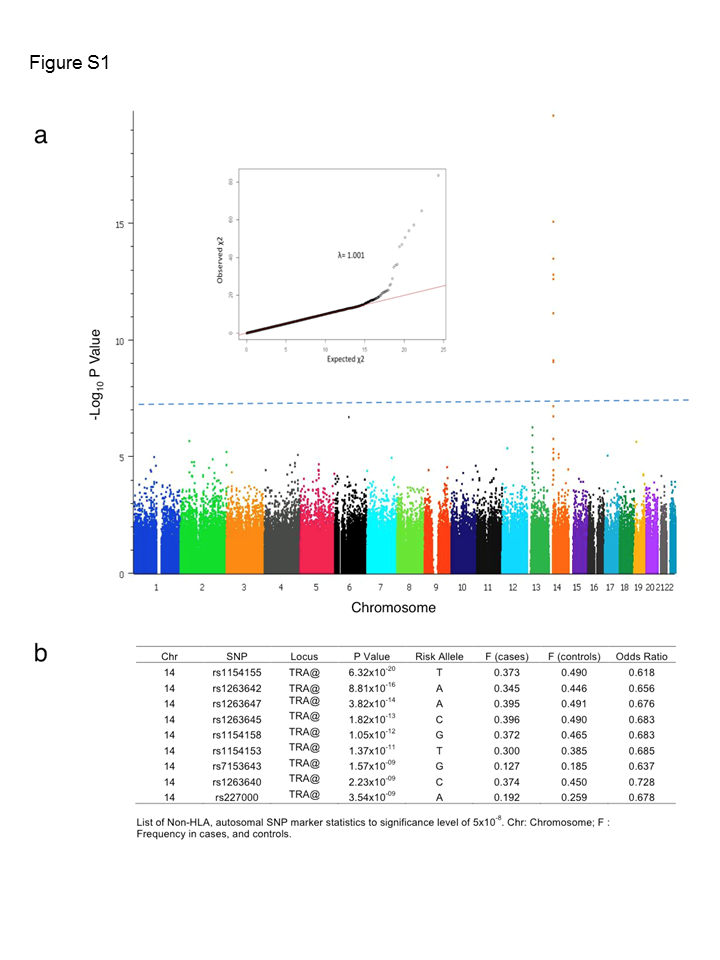

Supplement: Figure S1 — A: Plot of association statistics for 603,382 autosomal, non-HLA variants calculated with EMMAX. The significance threshold used (blue line) was P = 5×10^−8^. The inset depicts a quantile quantile plot of results observed (black circles), and slopes of estimated (red) versus expected chi square values (black line, invisible). The inflation statistic for tested markers is 1.001. B: List of risk variants to significance of P = 5×10^−8^. (TIF) [file pgen.1003880.s001.tif]

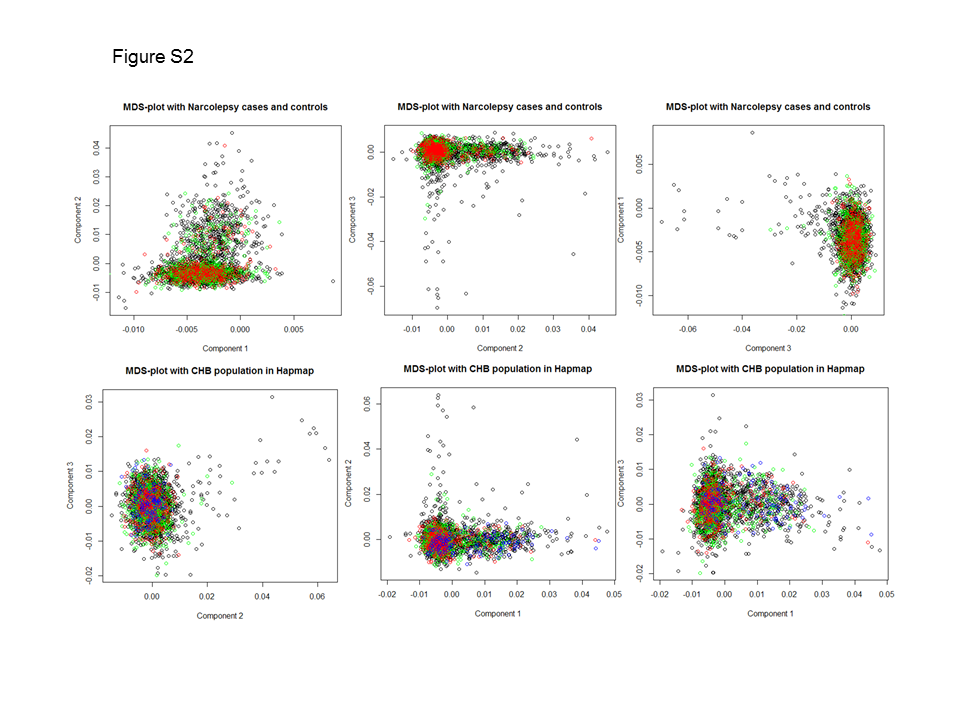

Supplement: Figure S2 — Multidimensional scaling plot of first three components in Chinese. The Chinese controls are shown in black, narcoleptics before 2009 shown in green, narcoleptics after 2009 in red and controls from Hapmap 3 in blue. (TIF) [file pgen.1003880.s002.tif]

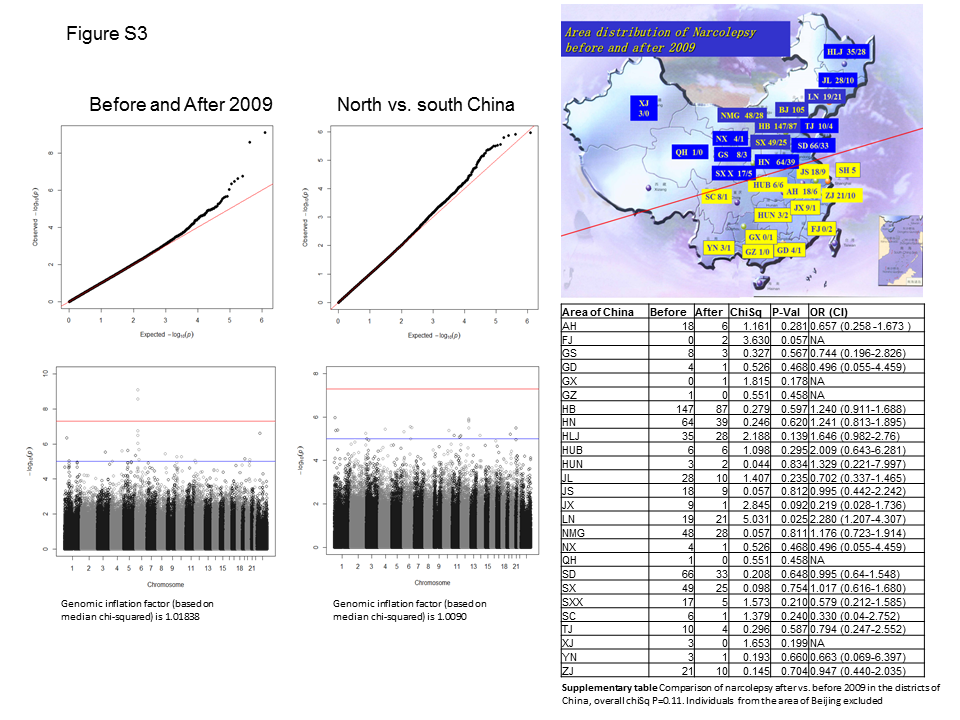

Supplement: Figure S3 — QQ-plot and Manhattan plot before and after 2009 and North vs. South China and comparison of individuals from the difference provinces in China for onset after vs. before 2009. (TIF) [file pgen.1003880.s003.tif]
